# Assessment of cefiderocol disk diffusion versus broth microdilution results when tested against *Acinetobacter baumannii* complex clinical isolates

**DOI:** 10.1128/spectrum.05355-22

**Published:** 2023-10-19

**Authors:** Yanling Liu, Li Ding, Renru Han, Lingbing Zeng, Junming Li, Yan Guo, Fupin Hu

**Affiliations:** 1 Institute of Antibiotics, Huashan Hospital, Fudan University, Shanghai, China; 2 Department of Clinical Laboratory, The First Affiliated Hospital of Nanchang University, Nanchang, China; 3 Key Laboratory of Clinical Pharmacology of Antibiotics, Ministry of Health, Shanghai, China; Emory University School of Medicine, Atlanta, Georgia, USA

**Keywords:** cefiderocol, *Acinetobacter baumannii*, disk diffusion, broth microdilution, categorical agreement, very major errors

## Abstract

The aim of this study was to evaluate the correlation between inhibitory zones and minimum inhibitory concentrations (MICs) when testing cefiderocol against *Acinetobacter baumannii* complex using disk diffusion and the broth microdilution (BMD) method according to the Clinical and Laboratory Standards Institute (CLSI) and European Committee on Antimicrobial Susceptibility Testing (EUCAST) criteria. Four-hundred and sixty-eight non-duplicated *A. baumannii* complex clinical isolates were collected from 56 hospitals of the China Antimicrobial Surveillance Network from 2019 to 2021. BMD using iron-depleted cation-adjusted Mueller-Hinton broth (ID-CAMHB) and standard disk diffusion methods were performed according to CLSI guidelines. Results were interpreted according to the CLSI and EUCAST breakpoints. Categorical agreement (CA), minor error (mE), major error (ME), and very major error (VME) were calculated for disk diffusion methods. The susceptibilities of all *A. baumannii* complex isolates by BMD were 98.7% (CLSI) and 97.6% (EUCAST). For all *A. baumannii* complex isolates, CA was 98.1% (CLSI) and 97.0% (EUCAST), with 0.9% (CLSI) and 1.9% (EUCAST) of VME, respectively. For the carbapenem-susceptible *A. baumannii* complex, the CA was 100%, with no mE or VME using both CLSI and EUCAST breakpoints. For carbapenem-resistant *A. baumannii* complex, CA was 97.5% (CLSI) and 96.2% (EUCAST), with 1.1% (CLSI) and 2.5% (EUCAST) of VME, respectively. Regarding the difficult-to-treat resistance *A. baumannii* complex isolates, CA was 97.6% (CLSI) and 95.7% (EUCAST), with 1.2% (CLSI) and 3.1% (EUCAST) of VME, respectively. Cefiderocol disk diffusion was difficult to assess in this study. Very few isolates were resistant to cefiderocol by BMD using CLSI breakpoint, and these were categorized as susceptible with the disk diffusion test. This study did, however, show that the main proportion of *A. baumannii* isolates were susceptible to cefiderocol by BMD, including carbapenem-resistant *A. baumannii*.

**IMPORTANCE:**

Carbapenem-resistant *Acinetobacter baumannii* is a major global health concern due to its high prevalence and limited treatment options. Cefiderocol is the only novel Food and Drug Administration (FDA)-approved β-lactam agent for the salvage treatment of carbapenem-resistant *A. baumannii* infection. Currently, a commercial automated susceptibility testing panel of cefiderocol is unavailable. Both the preparation of iron-depleted cation-adjusted Mueller-Hinton broth and the performance of broth microdilution are cumbersome in routine microbiology laboratories. A disk diffusion method is convenient for cefiderocol antimicrobial susceptibility testing, but limited data are available specifically for *A. baumannii* clinical isolates. Moreover, the Clinical and Laboratory Standards Institute published revisions to the *A. baumannii* cefiderocol disk diffusion breakpoints in 2022. Hence, we evaluated the performance of cefiderocol disk diffusion compared with the reference BMD against *A. baumannii* clinical isolates, especially those with cefiderocol zone diameters ≤ 14 mm.

## INTRODUCTION

Infections caused by carbapenem-resistant Gram-negative organisms are a major global public health threat worldwide (1). Due to the limited options for effective treatment, the Centers for Disease Control and Prevention and the World Health Organization have identified carbapenem-resistant *Acinetobacter baumannii* as a priority pathogen for which the development of new antibiotics is urgently needed (2, 3). Cefiderocol is a novel siderophore cephalosporin that uses bacterial iron transport to facilitate cell entry and achieve high concentrations in the periplasmic space (4). The cephalosporin moiety then binds to penicillin-binding proteins and inhibits peptidoglycan synthesis, ultimately leading to bacterial cell death (5). Due to its unique mechanism of action, cefiderocol overcomes resistance mechanisms due to porin channel mutations and upregulated efflux pumps; it also has intrinsic stability against hydrolysis by Class A, B, C, and D β-lactamases (1, 5), giving cefiderocol a potent activity against a wide range of carbapenem-resistant Gram-negative organisms, including both *Enterobacterales*, *Pseudomonas aeruginosa*, and *A. baumannii* complex (6, 7). Cefiderocol is currently approved by the U.S. Food and Drug Administration for the treatment of urinary tract infections, hospital-acquired pneumonia, and ventilator-associated bacterial pneumonia in 2019 and by the European Medicines Agency for the treatment of infections caused by Gram-negative bacteria in adults with limited therapeutic options in 2020 (8, 9).

Accurate *in vitro* susceptibility testing of cefiderocol is key to successful treatment. The gold standard susceptibility test for cefiderocol is MIC determination by broth microdilution method in ID-CAMHB as recommended by Clinical and Laboratory Standards Institute (10) and European Committee on Antimicrobial Susceptibility Testing (11). However, this requirement is challenging as the preparation of ID-CAMHB is time-consuming and not suitable for routine performance in most clinical microbiology laboratories. Disk diffusion is considered to be a reliable and less expensive method for many other antibiotics and is probably the most practical method for routine use in clinical laboratories. In 2020, the CLSI approved cefiderocol disk diffusion breakpoints for the *A. baumannii* complex. A revision with a susceptible-only disk diffusion breakpoint of ≥15 mm and a disk diffusion cefiderocol for Group B was published in the M100 32nd edition (10). Conversely, EUCAST recommends the use of non-species-specific pharmacokinetic/pharmacodynamic (PK/PD) breakpoints for *A. baumannii* complex and the provision of disk correlates associated with the susceptible PK-PD breakpoint (11). Published studies evaluating the performance of cefiderocol disk diffusion against Gram-negative Bacilli have focused on *Enterobacterales*. Limited data are available specifically for clinical isolates of *A. baumannii* complex. Therefore, we evaluated the performance of disk diffusion compared to reference BMD against *A. baumannii* complex clinical isolates, including carbapenem-susceptible and carbapenem-resistant *A. baumannii* complex.

## RESULTS

### Antimicrobial susceptibility testing

Cefiderocol exhibits potent *in vitro* activity with relatively low MICs (MIC_50/90_, 0.25/1 mg/L) against *A. baumannii* complex clinical isolates, with MICs ranging from ≤0.03 to >64 mg/L. 98.7% (462/468) and 97.6% (457/468) of *A. baumannii* complex isolates were susceptible to cefiderocol by BMD, according to the CLSI and EUCAST breakpoints, respectively; similar susceptibilities of 99.4% (465/468, CLSI) and 98.9% (463/468, EUCAST) were observed by disk diffusion. All 104 carbapenem-susceptible *A. baumannii* complex isolates were 100% susceptible to cefiderocol regardless of the breakpoint criteria or the method used. When using EUCAST breakpoints, the cefiderocol susceptibilities of carbapenem-resistant *A. baumannii* complex isolates and difficult-to-treat resistance (DTR) *A. baumannii* complex isolates were slightly lower than those obtained using CLSI breakpoints (Table 1). Cefiderocol non-susceptible (MIC ≥8 mg/L) was observed in six isolates; these isolates were also resistant to meropenem, cefepime, and ciprofloxacin. Of the six cefiderocol non-susceptible isolates, two isolates were intermediate to cefiderocol with MICs of 8 mg/L, and four isolates were resistant to cefiderocol with MICs of 32 mg/L (*n* = 2) and >64 mg/L (*n* = 2) according to CLSI breakpoints. The susceptibility of *A. baumannii* complex isolates to other antimicrobial agents is shown in Table 2.

**TABLE 1:** Agreement and errors for disk diffusion compared with broth microdilution

Organism	No. of isolates	Cefiderocol (mg/L)			% with breakpoint		Agreement or error (%）							
		MIC range	MIC_50_	MIC_90_	CLSI	EUCAST^*a*^	CLSI				EUCAST^*b*^			
					S^*c*^	S	CA	mE	ME	VME	CA	mE	ME	VME
All isolates	468	≤0.03 to >64	0.25	1	98.7	97.6	98.1	0.4	NA^*d*^	0.9	97.0	NA	NA	1.9
Carbapenem-susceptible *A. baumannii* complex	104	≤0.03–2	0.125	0.5	100	100	100	0	NA	0	100	NA	NA	0
Carbapenem-resistant *A. baumannii* complex	364	≤0.03 to >64	0.5	1	98.4	97	97.5	0.5	NA	1.1	96.2	NA	NA	2.5
Difficult-to-treat resistance *A. baumannii* complex	254	≤0.03 to >64	0.5	1	98	96.1	97.6	0.8	NA	1.2	95.7	NA	NA	3.1

^*a*^ Non-species-specific PK/PD breakpoints of ≤2 mg/L for S and >2 mg/L for R that can be applied to *A. baumannii* complex.

^*b*^ EUCAST provided disk correlates associated with the susceptible PK/PD breakpoint for *A. baumannii* complex.

^*c*^ S, susceptible.

^*d*^ NA, not applicable.

**TABLE 2:** Susceptibility of *A. baumannii* complex isolates to antimicrobial agents

Isolates (no.)	Antimicrobial agent	MIC (mg/L)			CLSI breakpoints (%)		EUCAST breakpoints (%)	
		Range	MIC_50_	MIC_90_	R^*b*^	S^*c*^	R	S
All isolates (*n* = 468）	Imipenem	≤0.06 to >128	64	128	77.8	22.2	77.8	22.2
	Meropenem	≤0.03 to >64	64	>64	77.8	22.2	77.8	22.2
	Piperacillin-tazobactam	≤2 to >256	>256	>256	76.9	21.4	NA	NA
	Cefoperazone-sulbactam	≤1 to >128	128	>128	73.1	23.5	NA	NA
	Ceftazidime	≤0.25 to >32	>32	>32	76.1	22.6	NA	NA
	Cefepime	≤0.06 to >128	128	>128	76.1	21.2	NA	NA
	Amikacin	≤1 to >128	>128	>128	64.7	35	65.8	34.2
	Ciprofloxacin	≤0.06 to >8	>8	>8	76.1	23.3	76.7	2.1
	Levofloxacin	≤0.125 to >16	16	>16	73.9	24.1	76.1	23.3
	Trimethoprim-sulfamethoxazole	≤0.25 to >32	>32	>32	60.5	39.5	57.1	39.5
	Colistin	≤0.06 to >128	0.5	1	1.9	98.1	1.9	98.1
	Tigecycline^*a*^	≤0.06 to >32	1	2	3	90.2	3	90.2
Carbapenem-susceptible *A. baumannii* complex (*n* = 104）	Imipenem	≤0.06–1	0.25	0.5	0	100	0	100
	Meropenem	≤0.03–2	0.25	0.5	0	100	0	100
	Piperacillin-tazobactam	≤2 to >256	≤2	16	1.9	91.3	NA	NA
	Cefoperazone-sulbactam	≤1–32	2	8	0	97.1	NA	NA
	Ceftazidime	≤0.25 to >32	4	8	1.9	94.2	NA	NA
	Cefepime	≤0.06–64	2	4	1	94.2	NA	NA
	Amikacin	≤1–16	2	4	0	100	1	99
	Ciprofloxacin	≤0.06 to >8	0.25	0.5	3.8	95.2	4.8	8.7
	Levofloxacin	≤0.125 to >16	≤0.125	0.25	2.9	97.1	2.9	95.2
	Trimethoprim-sulfamethoxazole	≤0.25–8	≤0.25	0.5	2.9	97.1	2.9	97.1
	Colistin	0.125 to >128	0.5	2	3.8	96.2	3.8	96.2
	Tigecycline^*a*^	≤0.06–2	0.125	0.5	0	100	0	100
Carbapenem-resistant *A. baumannii* complex (*n* =364）	Imipenem	16 to >128	64	128	100	0	100	0
	Meropenem	16 to >64	64	>64	100	0	100	0
	Piperacillin-tazobactam	≤2 to >256	>256	>256	98.4	1.4	NA	NA
	Cefoperazone-sulbactam	≤1 to >128	128	>128	94	2.5	NA	NA
	Ceftazidime	≤0.25 to >32	>32	>32	97.3	2.2	NA	NA
	Cefepime	≤0.06 to >128	128	>128	97.5	0.3	NA	NA
	Amikacin	≤1 to >128	>128	>128	83.2	16.5	84.3	15.7
	Ciprofloxacin	≤0.06 to >8	>8	>8	96.7	2.7	97.3	0.3
	Levofloxacin	≤0.125 to >16	16	>16	94.2	3.3	97	2.7
	Trimethoprim-sulfamethoxazole	≤0.25 to >32	>32	>32	76.9	23.1	72.5	23.1
	Colistin	≤0.06 to >32	0.5	1	1.4	98.6	1.4	98.6
	Tigecycline^*a*^	≤0.06 to >32	1	4	3.8	87.4	3.8	87.4
Difficult-to-treat resistance *A. baumannii* complex (*n* = 254）	Imipenem	16 to >128	64	128	100	0	100	0
	Meropenem	16 to >64	64	>64	100	0	100	0
	Piperacillin-tazobactam	256 to >256	>256	>256	100	0	NA	NA
	Cefoperazone-sulbactam	32 to >128	128	>128	97.6	0	NA	NA
	Ceftazidime	16 to >32	>32	>32	99.6	0	NA	NA
	Cefepime	16 to >128	128	>128	98.8	0	NA	NA
	Amikacin	≤1 to >128	>128	>128	84.6	15.4	85.8	14.2
	Ciprofloxacin	>8	>8	>8	100	0	100	0
	Levofloxacin	4 to >16	16	>16	97.6	0	100	0
	Trimethoprim-sulfamethoxazole	≤0.25 to >32	>32	>32	78	22	73.2	22.4
	Colistin	0.125–1	0.5	1	0	100	0	100
	Tigecycline^*a*^	0.125–32	1	4	3.1	89.4	3.1	89.4

^*a*^ According to U.S. FDA breakpoints; S, susceptible; R, resistant.

^*b*^ R, resistant.

^*c*^ S, susceptible.

### Correlation between inhibitory zones and MICs

**Fig 1:**
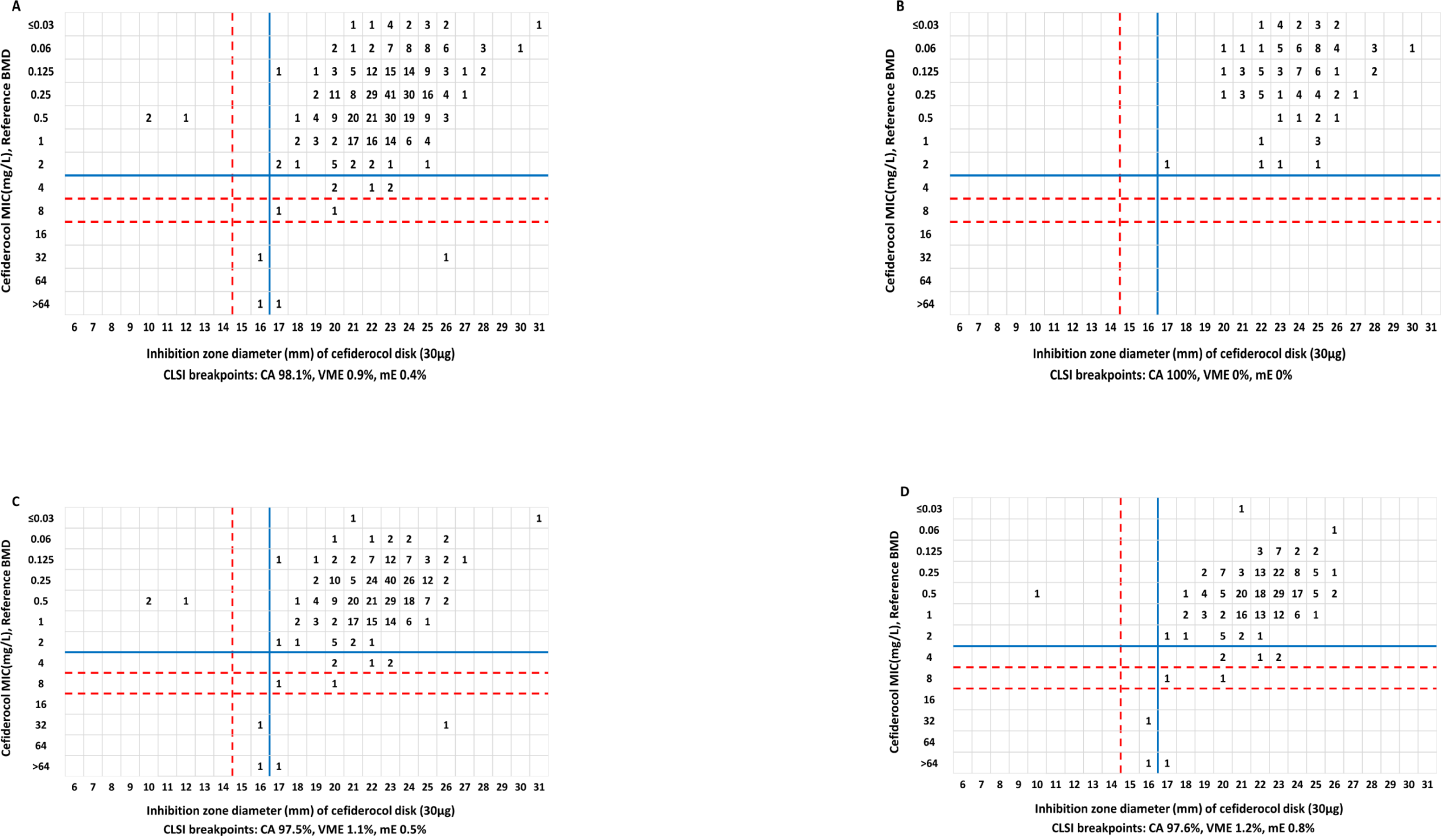
Scattergram comparing the results of cefiderocol BMD MIC values (mg/L) and disk diffusion zone diameters (mm) against *A. baumannii* complex. MICs for (**A**) all *A. baumannii* complex isolates (*n* = 468), (**B**) carbapenem-susceptible *A. baumannii* complex (*n* = 104), (**C**) carbapenem-resistant *A. baumannii* complex (*n* = 364), and (**D**) difficult-to-treat resistance *A. baumannii* complex (*n* = 254). Lines represent the applied breakpoints: red dashed line, CLSI breakpoints; blue, EUCAST PK/PD breakpoints.

The distribution of BMD cefiderocol MICs versus disk diffusion zone diameters is shown in Fig. 1. CA rates using CLSI and EUCAST breakpoints were 98.1% and 97.0%, respectively, for all *A. baumannii* complex isolates, 97.5% and 96.2% for carbapenem-resistant *A. baumannii* complex isolates, 97.6% and 95.7% for DTR *A. baumannii* complex isolates, and both 100% for carbapenem-susceptible *A. baumannii* complex isolates. The ME could not be calculated due to the absence of CLSI and EUCAST disk diffusion-resistant breakpoints for *A. baumannii* complex and the mE of the EUCAST criterion due to the absence of the MIC intermediate category. When tested against carbapenem-susceptible *A. baumannii* complex isolates, no mE or VME was observed using both CLSI and EUCAST breakpoints. CLSI breakpoints provided a higher correlation, with VMEs of 0.9% for all *A. baumannii* complex isolates, 1.1% for carbapenem-resistant *A. baumannii* complex, and 1.2% for DTR *A. baumannii* complex isolates. When the EUCAST breakpoints were applied, the VME reached 1.9% for all *A. baumannii* complex isolates, 2.5% for carbapenem-resistant *A. baumannii* complex isolates, and 3.1% for DTR *A. baumannii* complex isolates, none of which met the acceptance criteria.

Among the mEs observed for the disk diffusion when CLSI breakpoints were applied, both two isolates were intermediate by MIC value and were interpreted as susceptible category by the disk diffusion results. There were five carbapenem-resistant *A. baumannii* complex isolates, also described as DTR *A. baumannii* complex, with cefiderocol MICs of 4 mg/L that were susceptible by CLSI but resistant by EUCAST criteria. Of these five isolates, two isolates with zones of inhibition of 20 mm, two isolates with zones of inhibition of 23 mm, and one isolate with the zone of inhibition of 22 mm were all determined to be susceptible by disk diffusion regardless of CLSI or EUCAST breakpoint.

### The discrepancy between disk diffusion and BMD

**Fig 2:**
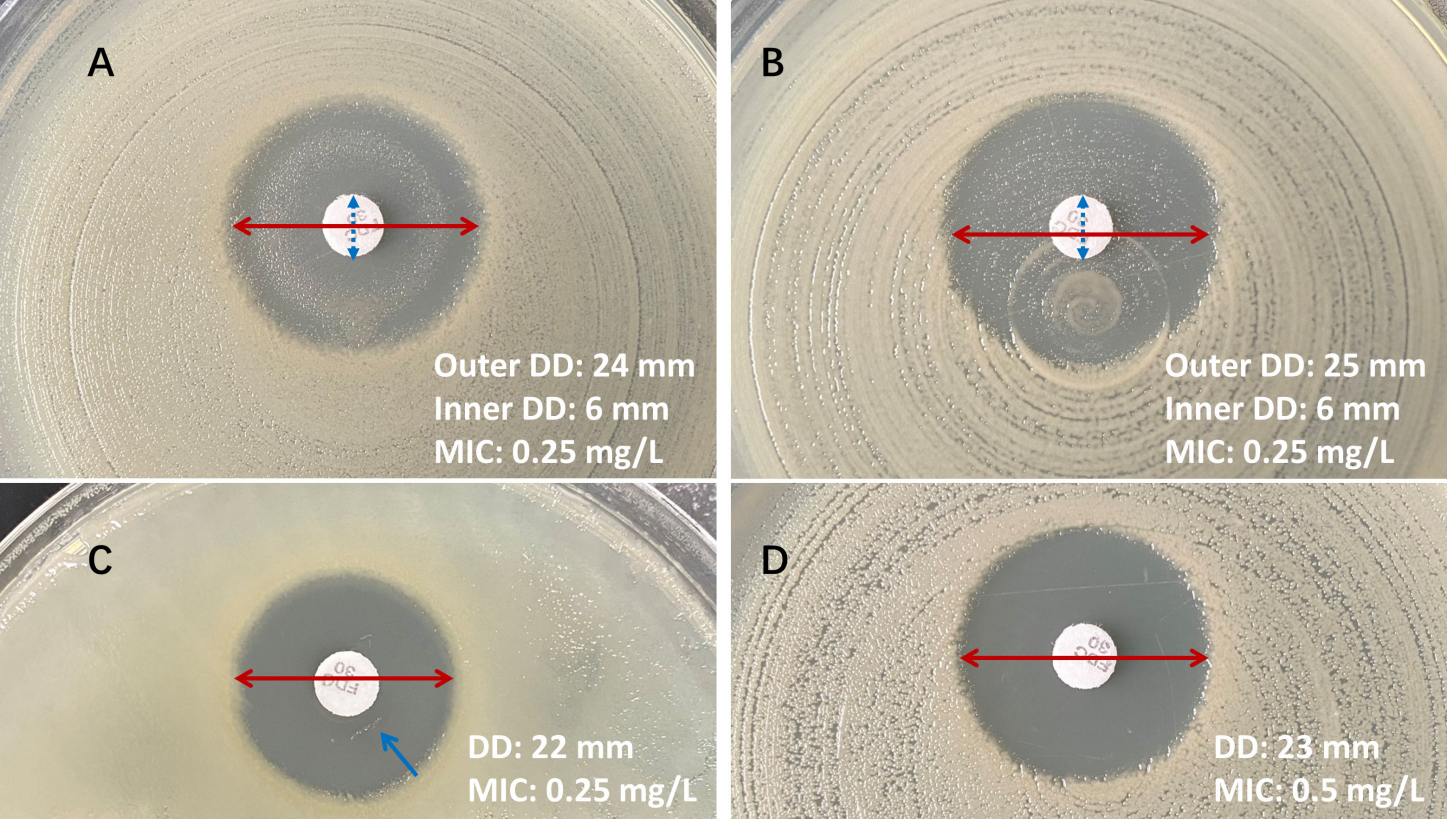
Disk diffusion (30 µg) interpretations of cefiderocol susceptibility testing. (**A**) Eagle phenomenon: significant growth of beach-like colonies within the inhibition zone, blue dashed arrow indicates the inner zone; (**B**) obviously isolated colonies within the inhibition zone; (**C**) ignoring the pinpoint colonies (blue arrow) within the inhibition zone; (**D**) clear inhibition zone.

There were only six isolates with cefiderocol MICs ≥ 8 mg/L in our study. All these isolates were classified as susceptible by disk diffusion when using CLSI breakpoints (Table 3). Of these six isolates, two isolates (MICs = 8 mg/L) had zone diameters of 17 and 20 mm, respectively; two isolates (MICs = 32 mg/L) had zone diameters of 16 and 26 mm, respectively; and two isolates (MICs > 64 mg/L) had zone diameters of 16 and 17 mm, respectively. Three carbapenem-resistant *A. baumannii* complex isolates with a disk diffusion zone diameter of ≤14 mm. Of these three isolates, one isolate with an inhibition zone of 12 mm and two isolates with inhibition zones of 10 mm were all confirmed as susceptible with MICs of 0.5 mg/L by BMD. Four isolates showed an Eagle effect, with significant growth of beach-like colonies or isolated colonies observed within the zone of inhibition (Fig. 2). All these isolates were confirmed as susceptible with MICs of 0.25 mg/L by BMD (Table 4). However, these four isolates showed distinct inner and outer zones and were recorded as the outer zone diameters.

**TABLE 3:** Isolates with discordance susceptibilities of cefiderocol between disk diffusion and broth microdilution

Isolate	MIC (mg/L)	Inhibitory zone (mm)
Cefiderocol non-susceptible^*a*^ (*n* = 6)		
ABA-00059	>64 (R)^*d*^	16 (S)^*b*^
ABA-00175	>64 (R)	17 (S)
ABA-00236	32 (R)	26 (S)
ABA-00281	32 (R)	16 (S)
ABA-00169	8 (I)^*c*^	17 (S)
ABA-00364	8 (I)	20 (S)
Zone diameter ≤ 14 mm (*n* = 3)		
ABA-00343	0.5 (S)	10 (NS)^*e*^
ABA-10086	0.5 (S)	10 (NS)
ABA-10063	0.5 (S)	12 (NS)

^*a*^ According to CLSI breakpoints.

^*b*^ S, susceptible.

^*c*^ I, intermediate.

^*d*^ R, resistant.

^*e*^ NS, non-susceptible.

**TABLE 4:** Isolates with Eagle effect^*a*^

Isolate	MIC (mg/L)	Outer inhibitory zone (mm)	Inner inhibitory zone (mm)
ABA-00039	0.25 (S)	22 (S)^*b*^	6 (NS)^*c*^
ABA-10059	0.25 (S)	24 (S)	6 (NS)
ABA-10064	0.25 (S)	25 (S)	6 (NS)
ABA-10066	0.25 (S)	24 (S)	6 (NS)

^*a*^ According to CLSI breakpoints.

^*b*^ S, susceptible.

^*c*^ NS, non-susceptible.

## DISCUSSION

Carbapenem-resistant *A. baumannii* complex is of particular global health concern due to its high prevalence and limited treatment options, which could cause an estimated 8,500 infections in hospitalized patients and 700 estimated deaths in the United States (2). The prevalence of carbapenem-resistant *A. baumannii* complex in China increased from 39% in 2005 to 76.6% in 2022 (12, 13). Although new β-lactam/β-lactamase inhibitor combinations such as ceftazidime-avibactam, ceftolozane-tazobactam, imipenem-relebactam, and meropenem-vaborbactam have recently been approved, none of these agents is active against carbapenem-resistant *A. baumannii* complex, which mainly produces Ambler Class D (OXA) β-lactamases (14). Cefiderocol is a parenteral siderophore cephalosporin, the only new β-lactam agent approved by FDA against carbapenem-resistant *A. baumannii* complex isolates (6). International surveillance reported that cefiderocol was active *in vitro* (MICs ≤ 4 mg/L) against 97.6% of carbapenem-non-susceptible *A. baumannii* complex collected from 2015 to 2016, with an MIC_50_ of 0.25 mg/L and MIC_90_ of 2 mg/L (1). Our data demonstrated that cefiderocol has potent *in vitro* activity against *A. baumannii* complex clinical isolates including carbapenem-resistant *A. baumannii* complex (susceptibility rate for 98.4%) and DTR *A. baumannii* complex (susceptibility rate for 98%).

Accurate MIC determination of cefiderocol requires the use of iron-depleted conditions to ensure the induction of ferric iron transporters (15). Since cefiderocol is currently not commercially available in an automated susceptibility testing panel and the preparation of ID-CAMHB and the performance of BMD are cumbersome, cefiderocol susceptibility testing poses a challenge to microbiology laboratories. Disk diffusion for cefiderocol (30 µg) was developed to be performed on standard Mueller-Hinton agar and does not require iron depletion, as the iron is sufficiently bound within the agar (16). Previous data have demonstrated that the disk diffusion method is a convenient alternative approach for cefiderocol susceptibility testing but performs poorly for *A. baumannii* (17 – 19). Our study showed that cefiderocol disk diffusion performed reasonably well when tested against carbapenem-susceptible *A. baumannii* complex isolates, with no mE or VME observed using both CLSI and EUCAST breakpoints. For all *A. baumannii* complex subgroups, both the CA and VME met the acceptance level according to CLSI breakpoints when cefiderocol disk diffusion results were compared with those of the BMD method. A previous study showed that the cefiderocol disk diffusion method gave a clinical categorization with 23.3% VME and 4.9% ME that did not meet the criteria when tested against CRE using the EUCAST breakpoints (18). Consistently, when EUCAST breakpoints were applied, CA met the acceptance level but VME did not meet in any *A. baumannii* complex subgroup in our study. Disk diffusion may not be able to detect the intermediate or resistant isolates based on our limited data, similar to those previously described for the *A. baumannii* complex (17, 18).

Reading cefiderocol MIC endpoints and measuring disk diffusion zone diameter can be challenging (10). Reports have shown that for some bacteria, the MIC determination by cefiderocol BMD may be trailed, making the determination of MIC endpoints difficult, especially for *A. baumannii* (17, 20). Both CLSI and EUCAST guidelines have highlighted the specific reading instructions of the cefiderocol BMD test; the MIC is read as the first well in which the significant reduction in growth corresponds to a button of <1 mm or is replaced by the presence of light or faint turbidity. In the present study, trailing endpoints or endpoints with slight turbidity growth were observed in five isolates. All of these isolates were susceptible to cefiderocol with low MICs, consistent with the disk diffusion results. A comparative study showed that disk diffusion was also problematic in cefiderocol susceptibility testing for the *A. baumannii* complex, which often had pinpoint colonies within the zones (17). Pinpoint colonies occurred in four carbapenem-resistant *A. baumannii* complex isolates in our study. According to the MIC value, we recommend ignoring pinpoint colonies and measuring the outer edge of the inhibition zone. In addition, differences in interpretation criteria established by CLSI and EUCAST could lead to considerable variability in results when interpreting cefiderocol antimicrobial susceptibility testing. For detailed guidance on the interpretation of cefiderocol disk diffusion and BMD results, we prefer the CLSI breakpoints based on our data with the lower VME, in line with a previous study (21).

CLSI M100 32nd revised the cefiderocol disk diffusion breakpoints for the *A. baumannii* complex, emphasizing that isolates with zone diameters ≤ 14 mm should not be reported without performing a MIC testing. Zone diameters ≤ 14 mm occur in resistant, intermediate, and susceptible isolates (10). In our study, all these isolates were confirmed as susceptible to cefiderocol with low MICs by BMD. When tested by the disk diffusion method, the Eagle effect and a narrow zone of inhibition with a fuzzy zone edge were observed. Four isolates with MICs of 0.25 mg/L showed the Eagle effect, i.e., significant growth of beach-like colonies or obvious colonies within the inhibition zone. Of note, all of these isolates showed distinct inner and outer zones in the disc diffusion results. As detailed guidelines for reading the cefiderocol disc result are not currently available, it is uncertain how to measure the zone diameter when the Eagle effect occurs. According to the cefiderocol BMD results in our study, all these four isolates were confirmed as susceptible with MICs of 0.25 mg/L. Therefore, we suggest that it is more reasonable to measure the outer zone diameter when the Eagle effect occurs (Fig. 2). The Eagle effect describes the phenomenon that bacteria exposed to concentrations of antibiotics above an optimal bactericidal concentration (OBC) have paradoxically improved levels of survival and has been reported for cell-wall synthesis inhibitors, including carbapenems and cephalosporins (22). This probably means that the OBC range of cefiderocol against the *A. baumannii* complex is in a relatively narrow space. Overall, MIC determination should be performed for isolates with zone diameters ≤ 14 mm or with difficult-to-measure disk diffusion results to avoid reporting false-susceptible or false-resistant.

This study has two limitations. One is that the experiment was conducted in a single laboratory rather than in a multicenter setting. The second is that the majority of strains in this study were susceptible to cefiderocol with fewer resistant strains, and cefiderocol disk diffusion was difficult to assess in this study. Very few isolates were resistant to cefiderocol by BMD using CLSI breakpoints, and these were categorized as susceptible with the disk diffusion test. This study did, however, show that the main proportion of *A. baumannii* tested were susceptible to cefiderocol by BMD, including carbapenem-resistant *A. baumannii*. As cefiderocol is not currently available in any commercial automated AST panel, disk diffusion may be a suitable alternative method for *A. baumannii* complex susceptibility testing to cefiderocol in clinical microbiology laboratories.

## MATERIALS AND METHODS

### Clinical isolates

A total of 468 non-duplicate *A. baumannii* complex clinical isolates, including 104 carbapenem-susceptible *A. baumannii* complex and 364 carbapenem-resistant *A. baumannii* complex, were collected from 56 hospitals from the China Antimicrobial Surveillance Network (CHINET, www.chinets.com) from January 2019 to October 2021. These clinical isolates were obtained from sputum (66.5%), followed by bronchial-alveolar lavage fluid (10.4%), blood (6%), urine (2.9%), shunt fluid (2.8%), secretions (2.6%), a catheter (2.1%), and other sources (6.7%). Isolates were identified by matrix-assisted laser desorption ionization-time-of-flight mass spectrometry (bioMérieux, France). The *Escherichia coli* ATCC 25922 and *P. aeruginosa* ATCC 27853 were used for quality control and antimicrobial susceptibility testing.

### Antimicrobial susceptibility testing

Antimicrobial susceptibility testing of cefiderocol was performed in parallel from the same inoculum suspension with disk diffusion (30 µg; MAST, Bootle, UK) on regular Mueller-Hinton agar (Oxoid, UK) and BMD in ID-CAMHB according to the CLSI reference method (10). Other antimicrobial susceptibility testing was performed using the standard BMD with the same bacterial inoculum according to the CLSI guidelines. The MICs and inhibition zone diameters of cefiderocol were interpreted according to the EUCAST PK/PD and CLSI breakpoints (CLSI: MIC ≤4 mg/L or zone diameter ≥15 mm as susceptible and MIC ≥16 mg/L as resistant; EUCAST: MIC ≤2 mg/L or zone diameter ≥17 mm as susceptible and MIC＞2 mg/L as resistant) (10, 11). According to the cefiderocol results in our study, when internal colonies occurred, if the colonies were significant or obvious growth, measure the outer zone diameter. Pinpoint colonies should be ignored and the outer edge of the inhibition zone measured.

### Definition

Carbapenem-resistant *A. baumannii* complex was defined as resistance to either imipenem or meropenem or both. The isolate was resistant to all β-lactams, including carbapenems and β-lactam/β-lactamase inhibitor combinations, and fluoroquinolones, which can be described as DTR isolate (23).

### Data analysis

CA indicated that the interpretation category results for the disk diffusion method were the same as those for the reference BMD using CLSI or EUCAST breakpoints (CA should be greater than 90%). VME indicated that the isolate was susceptible by disk diffusion but resistant by BMD. ME indicated that the isolate was resistant to disk diffusion but susceptible according to the BMD. mE indicated that the isolate was susceptible or resistant by disk diffusion but intermediate by BMD. Very major discrepancy rates should be less than 1.5%, and major discrepancy rates should be less than 3% when calculated based on all isolates according to CLSI M23-A5 (24).
